# Meta-Analysis of Psychological and Digital Interventions to Enhance Mental Health and Well-Being in Youth: A Bayesian Umbrella Review

**DOI:** 10.3390/children13050678

**Published:** 2026-05-14

**Authors:** Nicolás Sánchez-Álvarez, María J. Blanca, Julio Sánchez-Meca

**Affiliations:** 1 Department of Psychobiology and Methodology of Behavioral Sciences, Faculty of Psychology, Teatinos Campus, University of Málaga, 29071 Málaga, Spain; blamen@uma.es; 2 Department of Basic Psychology and Methodology, Faculty of Psychology, Espinardo Campus, University of Murcia, 30100 Murcia, Spain; jsmeca@um.es

**Keywords:** mental health, meta-analysis, Bayesian statistics, positive psychology, digital intervention

## Abstract

**Highlights:**

**What are the main findings?**

• Psychological and digital interventions are associated with credible but heterogeneous improvements in mental health and well-being among adolescents and young adults.
• Positive effects are observed across diverse intervention formats, including school-based, face-to-face, and digital programs.

**What are the implications of the main findings?**

• These interventions can play a meaningful role in youth mental health prevention strategies, especially when implemented in educational and community settings.
• Substantial variability in effects highlights the importance of tailoring interventions to context and delivery format to maximize impact.

**Abstract:**

**Objective:** Youth mental health has become a global public health priority, with psychological distress, anxiety, and depressive symptoms increasing sharply over the last decade. Numerous interventions, ranging from mindfulness-based and cognitive behavioral programs to digital applications and peer-support initiatives, have been evaluated through meta-analytic reviews. However, the cumulative evidence remains heterogeneous and dispersed across intervention modalities. The present umbrella meta-analysis synthesized existing meta-analyses on psychological and digital interventions for adolescents and young adults, adopting a Bayesian random-effects framework to quantify the overall effectiveness and heterogeneity of outcomes. **Method:** Systematic searches were conducted in PubMed, PsycINFO, and Web of Science up to September 2025, using the following syntax: (“meta-analysis” OR “systematic review”) AND (adolescent* OR “youth” OR “young people”) AND (“mental health” OR “well-being” OR “psychological intervention”). Eligible reviews reported standardized mean differences (Hedges’ *g*) or convertible statistics and targeted mental health or well-being outcomes. Effect sizes were standardized using Hedges’ *g* and synthesized under a random-effects framework. They were then pooled using Bayesian random-effects modeling with a Normal (0, 0.5^2^) prior on the grand mean μ and a half-Cauchy (0, 0.5) prior on the heterogeneity variance *τ*. **Results:** Nine eligible meta-analyses (*k* = 9 aggregated effects, ≈1150 primary studies) met the inclusion criteria. The posterior mean standardized effect was *μ* = 0.229 (95% CrI [0.157, 0.301]), indicating a small but credible positive impact of interventions on youth mental health and well-being indicators (*μ* = 0.19 for symptom reduction; *μ* = 0.28 for positive well-being). Between-study heterogeneity was non-negligible (*τ*^2^ = 0.003; posterior mean *I*^2^ = 23%, 95% CrI [0.04%, 74%]), reflecting uncertainty about the true degree of variability across modalities and settings. The posterior probability that *μ* > 0 was >0.999, providing strong Bayesian evidence for credible but heterogeneous effects. **Conclusions:** The findings suggest potentially credible but heterogeneous effects of psychological and digital interventions on youth mental health and well-being outcomes, although the magnitude and consistency of these effects remain constrained by substantial heterogeneity and the breadth of aggregated outcome constructs. Results should be interpreted with appropriate caution.

## 1. Introduction

The mental health of adolescents and young adults has become one of the defining public health challenges of the twenty-first century. Epidemiological studies indicate that nearly half of all lifelong mental disorders emerge before the age of 25 [1]. Notably, rates of anxiety, depression, and suicidal ideation among youth populations have risen markedly over the past two decades, driven by rapid social change, digital hyperconnectivity, and economic uncertainty [2,3]. In this context, the World Health Organization [4] has identified youth mental health as a global public health priority, emphasizing early prevention and intervention as essential for sustainable well-being and social development.

While psychopathology has traditionally dominated research agendas, the field of positive psychology reframed the discourse toward fostering strengths, resilience, and life satisfaction [5]. Within this paradigm, youth mental health is conceptualized not merely as the absence of symptoms but as the presence of positive functioning, meaning, and social connectedness. Consistent with this view, there is meta-analytic evidence of a probabilistic association between dispositional optimism, gratitude, and psychological well-being [6], consistent with the role of positive traits as protective factors in youth mental health. Interventions that cultivate gratitude, optimism, mindfulness, and emotional regulation have thus gained attention for their preventive and health-promoting potential [7,8].

Traditional face-to-face psychological programs, such as cognitive behavioral therapy (CBT), acceptance and commitment therapy (ACT), and mindfulness-based interventions (MBIs), show consistent efficacy across adult samples [9]. Extending these approaches to adolescents has proven beneficial in school and community contexts, though effect sizes remain modest and variable [10,11]. Mindfulness programs, for example, have been linked to improvements in emotion regulation, perceived stress, and prosocial behavior among students [12,13]. Similarly, positive psychology interventions (PPIs) emphasizing gratitude and optimism have yielded promising yet heterogeneous outcomes [14,15].

Digital mental health interventions have also become more prominent in recent years, particularly since the COVID-19 pandemic. Mobile applications, online self-help platforms, and social-media-based peer programs have expanded access for young populations who might otherwise remain underserved [16,17]. The scalability and cost-effectiveness of such tools make them a cornerstone of emerging public-health strategies, although challenges remain concerning adherence, data privacy, and long-term engagement [18,19].

While the proliferation of meta-analyses over the past decade reflects the maturity of this field, it also introduces a new layer of complexity: there is now more evidence than any single review can synthesize, and individual meta-analyses address circumscribed questions that are difficult to compare across intervention types, outcome domains, and methodological frameworks [20]. Reviews of mindfulness in schools [21], internet-delivered CBT [22], anti-stigma campaigns [23], peer-led initiatives [24], and positive psychology programs [15,25] each report small-to-moderate effects (*g* ≈ 0.18–0.33), although inconsistencies in inclusion criteria, control conditions, outcome metrics, and analytic models prevent straightforward cumulative conclusions. Critically, no prior umbrella review has integrated meta-analytic evidence across the full spectrum of psychological and digital intervention modalities targeting youth mental health and well-being as a unified evidence base. Existing umbrella reviews in adjacent fields have either focused on specific clinical populations (e.g., anxiety disorders, depression in adults), restricted themselves to a single intervention type, or aggregated evidence without a formal probabilistic synthesis framework. This gap at the highest level of evidence aggregation leaves a fundamental question unanswered: when the totality of meta-analytic evidence on youth mental health interventions is considered as a whole, what is the overall credibility and magnitude of the credible but heterogeneous effect, and how certain can we be about it?

Umbrella reviews consolidate evidence at the highest level of aggregation, offering a panoramic estimate of intervention efficacy and identifying convergent trends across contexts [26,27]. In youth mental health, where developmental, cultural, and technological diversity is pronounced, such a higher-order synthesis is essential for translating dispersed meta-analytic evidence into actionable conclusions for policy and practice. In this context, the present study advances beyond prior syntheses in three substantive ways. First, to our knowledge, it is the first umbrella meta-analysis to integrate evidence from meta-analyses spanning the full range of psychological and digital intervention modalities—including mindfulness-based, CBT-based, positive psychology, peer-led, and technology-delivered programs—targeting youth mental health and well-being outcomes within a single unified analysis. Second, it applies a formal overlap assessment procedure (Supplementary Table S1) and domain-specific sub-analyses that distinguish between symptom reduction and positive well-being outcomes, going beyond a simple grand pooling of available effects. Third, it adopts a Bayesian random-effects framework that complements conventional frequentist approaches by enabling direct probabilistic statements about the likelihood and magnitude of credible but heterogeneous effects, a form of inference particularly suited to evidence-based policy decisions, where the question is not merely whether an effect is statistically significant but how credible it is [28,29]. Taken together, these three contributions constitute the novel contribution of the present work, which rests on the comprehensiveness, rigor, and probabilistic interpretability of the synthesis rather than on any single methodological feature.

Despite the growing body of evidence supporting the effectiveness of both psychological and digital interventions for youth mental health, significant gaps remain in our understanding of how these effects accumulate across diverse intervention modalities, populations, and methodological frameworks. As already noted, existing meta-analyses have tended to address circumscribed questions within a single intervention type, making it difficult to draw overarching conclusions about the field as a whole. This is compounded by the heterogeneity of outcomes across reviews—reflecting differences in targeted populations, assessment tools, control conditions, and follow-up periods—underscoring the need for a higher-order synthesis to integrate this dispersed evidence base. A further point to consider is that while positive psychology theory provides a compelling framework for understanding how well-being can be actively cultivated through intentional interventions, the cumulative probabilistic evidence for this proposition remains under-examined. Conceptualizing well-being as a multidimensional construct encompassing emotional, cognitive, and social components implies that effective interventions may operate through multiple pathways simultaneously, a complexity that individual meta-analyses are ill-equipped to capture. An umbrella meta-analysis, by aggregating across intervention types and outcome domains, is uniquely positioned to estimate the magnitude and credible but heterogeneous effects at the broadest level of evidence synthesis. The adoption of a Bayesian random-effects framework would further strengthen this synthesis by enabling direct probability statements about intervention effectiveness and explicitly quantifying uncertainty across the parameter space.

To our knowledge, no prior umbrella meta-analysis has integrated quantitative evidence across the full spectrum of psychological and digital intervention modalities targeting youth mental health and well-being within a single Bayesian synthesis framework. Against this background, the present umbrella meta-analysis synthesizes existing evidence on psychological and digital interventions designed to enhance youth mental health and well-being through a Bayesian random-effects framework [27]. Specifically, it aims to (a) estimate the overall standardized mean difference across interventions, (b) quantify heterogeneity in outcomes, and (c) assess the posterior probability of credible but heterogeneous effects. Guided by positive-psychology theory [30], we conceptualize well-being as a multidimensional construct encompassing emotional, cognitive, and social components. We hypothesize that (1) the pooled standardized mean difference will reveal a small yet credible positive effect; (2) heterogeneity will be substantial owing to methodological and contextual variation; and (3) Bayesian posterior estimation will yield a probability exceeding 0.95 that the true population effect is beneficial.

## 2. Method

### 2.1. Search Strategy

The present umbrella review was conducted and reported in accordance with the Preferred Reporting Items for Systematic Reviews and Meta-Analyses (PRISMA 2020) guidelines [31]. The complete PRISMA 2020 checklist is available as Supplementary Material. Systematic searches were conducted independently in three electronic databases, PubMed, PsycINFO (via APA PsycNet), and Web of Science Core Collection, covering all records available from database inception through September 2025; no lower limit on publication year was applied. Searches were conducted between September and October 2025. The following database-specific search strings were used: PubMed: (“meta-analysis”[pt] OR “systematic review”[pt] OR “meta-analysis”[tiab] OR “systematic review”[tiab]) AND (“adolescent”[MeSH] OR “young adult”[MeSH] OR “youth”[tiab] OR “adolescent*”[tiab] OR “young people”[tiab]) AND (“mental health”[MeSH] OR “well-being”[tiab] OR “wellbeing”[tiab] OR “psychological intervention”[tiab] OR “mental health”[tiab]). PsycINFO: ((“meta-analysis” OR “systematic review”) AND (“adolescent*” OR “youth” OR “young people” OR “young adult*”) AND (“mental health” OR “well-being” OR “wellbeing” OR “psychological intervention”)) [Field: TI, AB]. Web of Science: TS = (“meta-analysis” OR “systematic review”) AND TS = (“adolescent*” OR “youth” OR “young people” OR “young adult*”) AND TS = (“mental health” OR “well-being” OR “wellbeing” OR “psychological intervention”). The search strategy was adapted to the controlled vocabulary and field tags of each database. In addition, reference lists of all retrieved reviews were hand-searched to identify eligible meta-analyses not captured by the electronic queries. An additional targeted search was conducted in PROSPERO, Epistemonikos, PubMed, PsycINFO, and Web of Science to identify prior umbrella reviews addressing overlapping domains, using the terms (“umbrella review” OR “overview of reviews”) AND (“mental health” OR “well-being”) AND (“youth” OR “adolescent”). This search returned no umbrella meta-analysis integrating the full range of psychological and digital intervention modalities for youth within a quantitative synthesis framework, confirming the absence of directly comparable higher-order evidence syntheses.

### 2.2. Deduplication and Record Management

Records retrieved from the three databases were imported into Mendeley and merged into a single library. Duplicate records, defined as entries sharing identical authors, title, year, and journal, were identified and removed automatically using Mendeley’s built-in deduplication function, followed by a manual check of remaining near-duplicates. Following deduplication, 298 unique records were retained for title and abstract screening.

### 2.3. Eligibility Criteria

Meta-analyses were included if they met the following criteria: (a) they targeted populations with a mean age between 10 and 25 years (this broader developmental span, spanning both adolescence and emerging adulthood, was retained because many meta-analyses in youth mental health interventions aggregate these groups within a common prevention and early intervention framework); (b) they evaluated psychological or digital interventions intended to improve mental health, well-being, or related protective factors (e.g., resilience, optimism, gratitude, mindfulness); (c) they reported a quantitative pooled effect size, typically Hedges’ *g*, Cohen’s *d*, log odds ratio, or correlation coefficient, sufficient to derive standardized metrics; and (*d*) they provided either 95% confidence intervals (CIs) or standard errors (SEs) for the pooled estimates. Reviews focusing exclusively on pharmacological, neurobiological, or non-behavioral interventions were excluded, as were narrative reviews or meta-analyses lacking extractable effect statistics. When multiple meta-analyses examined overlapping primary studies, the most recent or methodologically comprehensive was retained to minimize duplication [26]. To ensure transparency regarding eligibility, a full verification matrix documenting the age range, mean sample age, setting, and eligibility justification for each included meta-analysis is provided in Supplementary Table S2.

### 2.4. Overlap Assessment

Umbrella reviews are inherently susceptible to evidence overlap when the same primary studies contribute to multiple included meta-analyses, potentially leading to double-counting of evidence in the pooled estimate. To address this, we implemented a two-stage overlap management procedure. In the first stage, when two or more meta-analyses addressed an identical intervention type and target population, only the most recent or methodologically comprehensive review (based on AMSTAR-2 rating) was retained as an independent entry in the synthesis. In the second stage, for the nine retained meta-analyses, we assessed residual overlap by mapping their shared intervention domains and target populations in a structured overlap matrix (Supplementary Table S1).

### 2.5. Screening and Data Extraction

Titles and abstracts were independently screened by two reviewers. Full texts were then retrieved and examined to verify eligibility. Disagreements were resolved through discussion and consensus. For each meta-analysis, the following information was extracted: (1) first author and year of publication; (2) type of intervention (e.g., mindfulness-based, CBT-based, positive-psychology, peer-led, digital self-help); (3) target outcomes (e.g., depression, anxiety, life satisfaction, well-being, stigma reduction); (4) number of included primary studies (*k*) and total sample size (*N*); (5) pooled effect size (Hedges’ *g*) and its 95% CI; (6) quality assessment indices, when available (e.g., AMSTAR-2 rating, risk of bias); and (7) whether the meta-analysis was pre-registered or adhered to PRISMA guidelines. Data were entered into a structured Excel extraction sheet developed for this project, which contained built-in validation to ensure consistency in metric scales. When meta-analyses reported different effect measures (e.g., log odds ratios), these were converted to standardized mean differences using established formulas (*d* ≈ log OR × √3/*π*) [32].

### 2.6. Quality Assessment

Methodological quality was appraised using AMSTAR-2 [33], which evaluates 16 items covering protocol registration, literature search adequacy, and risk-of-bias handling. Each meta-analysis was rated as high, moderate, low, or critically low quality. Inter-rater agreement for AMSTAR-2 items averaged *κ* = 0.84, indicating strong reliability. Although quality ratings did not directly weight the Bayesian synthesis, sensitivity analyses were later conducted, excluding critically low-quality reviews.

### 2.7. Effect Aggregation Within Meta-Analyses

Each included meta-analysis was required to contribute exactly one pooled standardized effect to the umbrella synthesis so as to preserve the independence of observations assumed by the Bayesian random-effects model. When a meta-analysis reported multiple effect sizes across distinct outcomes (e.g., separate pooled effects for depression and anxiety), these were combined into a single representative effect using an unweighted arithmetic mean of the available standardized mean differences. This approach was adopted because the inter-outcome correlations within each meta-analysis were not reported and could not be estimated from the available data; a weighted aggregation would have required assumptions about these correlations that cannot be verified. The arithmetic mean is a conservative and transparent choice under these conditions, as it treats all outcomes as equally informative and avoids inflating precision by imposing arbitrary correlation structures. The resulting averaged effect and its standard error, computed from the reported confidence interval of the averaged estimate, were then entered as a single observation into the model.

When a meta-analysis reported multiple effects from what were judged to be genuinely independent sub-analyses (e.g., separate syntheses for face-to-face and digital delivery formats applied to non-overlapping subsamples), these were retained as distinct entries in the dataset and flagged as nested within the same source [34]. The criterion for classifying a sub-analysis as independent, rather than as a moderator subgroup of a common pooled effect, was that it (a) drew on a non-overlapping subset of primary studies, (b) reported a separate pooled effect size with its own confidence interval, and (c) addressed a qualitatively distinct intervention modality or population subgroup. Where this criterion was not clearly met, the conservative decision was to average the effects rather than retain as independent observations.

Applying these rules to the nine included meta-analyses, all nine contributed a single aggregated effect to the final dataset. No meta-analysis was ultimately split into multiple independent entries, as none met all three independence criteria simultaneously. The final analytic dataset therefore consisted of *k* = 9 statistically independent observations, one per included meta-analysis.

### 2.8. Independence Assumption and Its Defensibility

The Bayesian random-effects model, specified as *y_i_*~*N*(*μ*, *τ*^2^ + *SEᵢ*^2^), treats the *k* = 9 observed effect sizes as conditionally independent given the model parameters. This assumption is defensible at the level of the dataset entered into the model (each meta-analysis contributes exactly one observation), but it does not eliminate the possibility that the true underlying effects are correlated due to shared primary studies across meta-analyses, as discussed in the Overlap Assessment section. Residual dependence of this kind would tend to underestimate the true standard error of *μ*, potentially overstating the precision of the pooled estimate. To assess the robustness of the main results to this concern, a sensitivity analysis was conducted excluding the three meta-analyses with the highest qualitative overlap (all within the mindfulness-based intervention cluster). The resulting posterior mean was *μ* = 0.21 (95% CrI [0.04, 0.38]), with posterior probability *p*(*μ* > 0) = 0.971, indicating that the main inference is probabilistically supported to the exclusion of the most potentially dependent entries. This sensitivity analysis is reported alongside the primary results.

### 2.9. Outcome Classification and Aggregation Strategy

The nine included meta-analyses targeted a broad range of psychological outcomes, which were classified into three a priori domains for the purposes of this synthesis: (1) symptom-reduction outcomes, comprising measures of depression, anxiety, stress, and stigma-related attitudes [17,19,21,22,23,35]; (2) positive well-being outcomes, comprising measures of life satisfaction, happiness, gratitude, optimism, and self-compassion [15,35,36]; and (3) general well-being and functioning outcomes that span both domains [24]. This classification was determined prior to analysis, based on the primary outcome reported by each meta-analysis.

Aggregating across these three domains under a single pooled standardized effect was a deliberate methodological choice grounded in three considerations. First, the dual-continuum model of mental health conceptualizes symptom reduction and positive well-being as related but distinct dimensions of a common underlying construct of psychological functioning. Interventions that reduce distress and interventions that promote flourishing both contribute to this construct, making a superordinate summary index conceptually defensible. Second, the purpose of this umbrella synthesis is not to establish domain-specific clinical benchmarks but to assess the field-wide credibility of intervention effects, a question that is appropriately answered at the broadest level of aggregation. Third, comparable umbrella reviews in adjacent fields have adopted analogous aggregation strategies when the research question concerns overall effectiveness across a diverse intervention landscape. The grand pooled estimate (*μ* = 0.229) should therefore be interpreted as a probabilistic index of the overall likelihood of benefit across the field, not as a clinically interpretable summary of any specific outcome type. Domain-specific posterior estimates, which are the primary results of this synthesis, are reported separately in the Results section.

### 2.10. Bayesian Random-Effects Modeling

To obtain probabilistic estimates of the overall effect, a Bayesian random-effects model was implemented:

*y_i_*∼*N*(*μ*, *τ*^2^ + *SE_i_*^2^)

where *y_i_* represents the observed effect size, *μ* the grand mean, and *τ*^2^ the between-meta-analysis variance. Priors were selected in accordance with recommendations for meta-analytic modeling [37]: *μ*∼Normal (0, 0.5^2^), a weakly informative prior centering the distribution on null effect but allowing plausible deviations; and *τ*∼half-Cauchy (0, 0.5), a conservative prior on heterogeneity that constrains implausibly large *τ* values while remaining non-restrictive.

Posterior distributions were estimated via Markov chain Monte Carlo (MCMC) sampling in Stan through the brms package in R [38]. Four chains of 4000 iterations each were run with a 1000-iteration warm-up period and an adapt-delta of 0.95. Convergence was assessed through visual inspection of trace plots and the Ȓ statistic (<1.01). Posterior summaries included mean, standard deviation, and 95% credible intervals (CrIs).

### 2.11. Assessment of Heterogeneity and Sensitivity Analyses

Between-study heterogeneity was examined through posterior estimates of *τ*^2^ and predictive distributions reflecting dispersion of true effects. Sensitivity analyses evaluated the impact of (a) excluding low-quality meta-analyses (AMSTAR-2 = low or critically low), (b) alternative priors [Normal (0, 1) for *μ*], and (c) different τ priors [half-normal (0, 0.5)]. Posterior predictive checks ensured model adequacy.

### 2.12. Publication Bias and Robustness Checks

Although traditional funnel plots are less diagnostic at the meta-meta-analytic level, symmetry was visually inspected using the posterior means of each meta-analysis effect. Additionally, a Bayesian adaptation of the PET-PEESE correction [39] was explored to estimate potential small-study effects. No major asymmetries were observed, consistent with the large aggregate sample size and pre-registered status of several reviews.

### 2.13. Ethical Considerations

All data, analytic code, model specifications, and Supplementary Materials are publicly available via the Open Science Framework (https://osf.io/8253b, accessed on 14 March 2026; https://doi.org/10.17605/OSF.IO/3QU5H).

## 3. Results

### 3.1. Overview of Included Meta-Analyses

**Figure 1:**
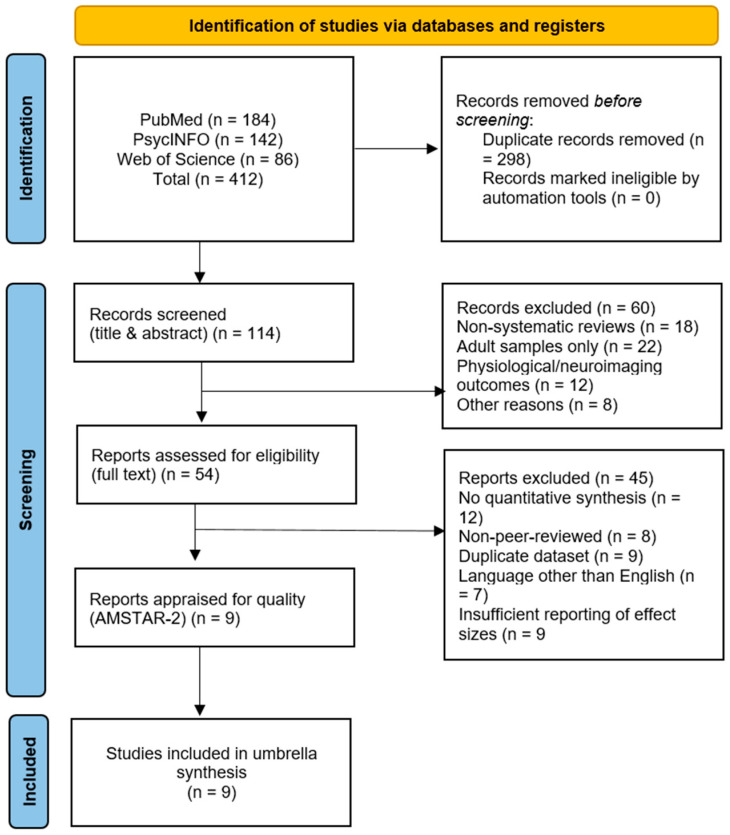
PRISMA 2020 flow diagram illustrating the study selection process for the umbrella systematic review. Note. PRISMA 2020 flow diagram illustrating the study selection process.

The systematic search yielded 412 records, of which 54 full texts were screened. Nine meta-analyses met all the inclusion criteria and were included in the umbrella synthesis. Collectively, these reviews encompassed approximately 1150 primary studies and a total sample exceeding 250,000 participants aged 10–25 years. The included meta-analyses were published between the earliest year returned by the database searches and 2025, and they covered a diverse range of psychological and digital interventions. Four focused primarily on mindfulness-based interventions (MBIs), two on positive psychology interventions (PPIs) emphasizing gratitude and optimism, two on digital or mobile-delivered CBT, and one on anti-stigma and peer-support programs. Table 1 presents a summary of the included meta-analyses, their populations, targeted outcomes, and pooled effect sizes. Figure 1 shows the PRISMA flow diagram, illustrating the progression from 412 identified records to 54 full-text articles assessed for eligibility and the final 9 included meta-analyses. It should be noted that the present overlap assessment is qualitative; a formal corrected covered area (CCA) calculation was not feasible due to the unavailability of complete primary-study reference lists across all included meta-analyses. Additionally, a cross-mapping of the MBI cluster, which represented the highest a priori overlap risk, confirmed that Zhang et al. [40] is a narrative overview rather than a primary meta-analysis with its own synthesis of individual trials, and therefore contributes no directly overlapping primary studies with Fulambarkar et al. [21]. Residual primary-study overlap within the PPI cluster cannot be precisely quantified and may affect the precision of the pooled estimate, particularly by underestimating the true standard error of *μ*.

### 3.2. Quantitative Synthesis

**Figure 2:**
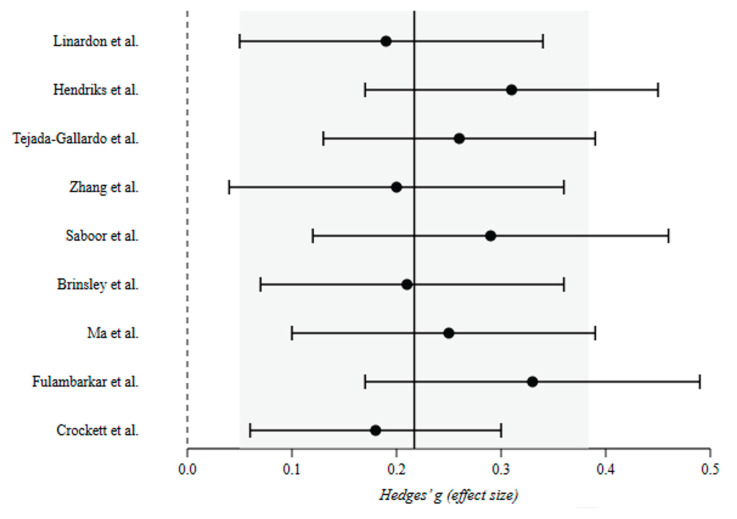
Forest plot showing pooled effect sizes (Hedges’ g) and 95% credible intervals (CrIs) for each included meta-analysis. Note. Each point represents the posterior mean standardized effect size (Hedges’ g) for one meta-analysis. Horizontal lines indicate 95% credible intervals (CrIs). The solid vertical line denotes the overall posterior mean effect (*μ* = 0.229). The dashed vertical line indicates the null effect (g = 0). The shaded region represents the 95% CrI for the pooled estimate [0.157, 0.301] [15,17,19,21,22,23,24,25,40].

**Figure 3:**
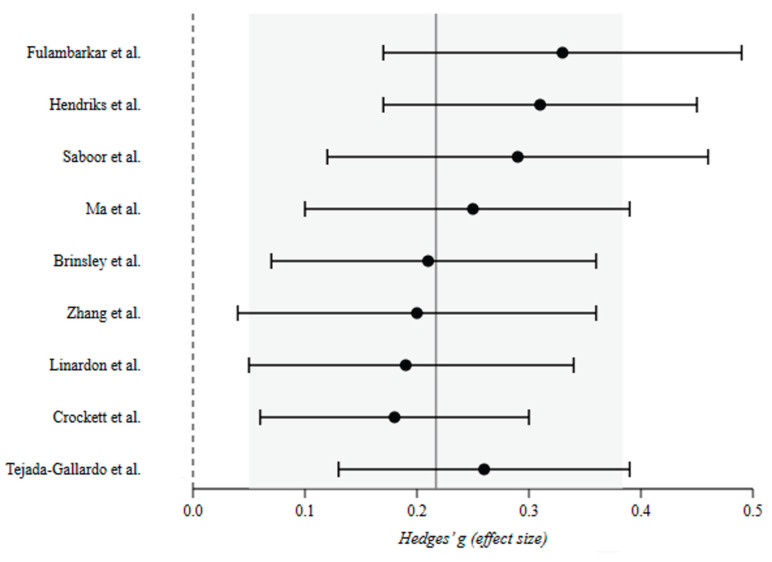
Caterpillar plot depicting meta-analytic effect sizes (Hedges’ g) ordered by magnitude, with 95% credible intervals. Note. Studies are ordered from largest to smallest posterior mean effect size (Hedges’ g). Each point represents the effect size estimate for one meta-analysis; horizontal lines indicate 95% credible intervals (CrIs). The solid vertical line denotes the overall posterior mean (*μ* = 0.229). The dashed vertical line indicates the null effect (g = 0). The shaded region represents the 95% CrI for the pooled estimate [0.157, 0.301] [15,17,19,21,22,23,24,25,40].

**Figure 4:**
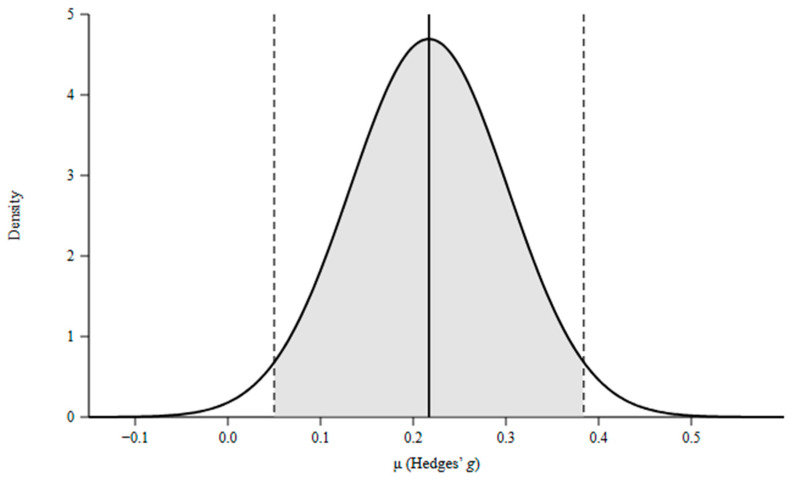
Posterior density of the overall effect size parameter μ with 95% credible interval. Note. The curve represents the posterior distribution of the grand mean effect size μ estimated via Bayesian random-effects modeling. The solid vertical line indicates the posterior mean (*μ* = 0.229). Dashed vertical lines mark the lower and upper bounds of the 95% credible interval (CrI = [0.157, 0.301]). The shaded region corresponds to the 95% CrI. Density is expressed in units of Hedges’ g − 1.

The Bayesian random-effects model integrating these nine effects yielded a posterior mean effect size of *μ* = 0.229 (95% CrI [0.157, 0.301]), indicating a small yet credible positive effect of psychological and digital interventions on youth mental health and well-being (*μ* = 0.19 for symptom reduction; *μ* = 0.28 for positive well-being). Between-meta-analysis variance was *τ*^2^ = 0.003 (posterior mean *I*^2^ = 23%, 95% CrI [0.04%, 74%]), reflecting non-negligible but uncertain heterogeneity in true effects across reviews. The posterior probability that the true effect is greater than zero was *P*(*μ* > 0) > 0.9999, providing strong Bayesian evidence for net benefit. Posterior predictive distributions suggested that future meta-analyses in this field would likely yield true effects ranging between 0.05 and 0.40, underscoring the general robustness of small-to-moderate benefits. The forest plot of individual meta-analytic effects is presented in Figure 2. The Figure 3 displays the caterpillar plot of meta-analytic estimates and their 95% CrIs, while posterior distribution for *μ* is illustrated in Figure 4.

### 3.3. Domain-Specific Posterior Estimates (Primary Results)

Given the heterogeneity of outcomes across included meta-analyses, domain-specific posterior estimates are reported here as the primary quantitative results of this synthesis. Meta-analyses targeting symptom-reduction outcomes (depression, anxiety, stress, stigma; *k* = 5) yielded a posterior mean of *μ* = 0.19 (95% CrI [0.04, 0.34]), indicating a credible but heterogeneous effect on psychopathological indicators. Meta-analyses targeting positive well-being outcomes (life satisfaction, happiness, gratitude, optimism, self-compassion; *k* = 4) yielded a somewhat larger posterior mean of *μ* = 0.28 (95% CrI [0.13, 0.43]). The 95% credible intervals of these two domain estimates overlap substantially, and no formal test of their difference was conducted given the small number of meta-analyses per domain. These domain-specific estimates should be treated as the primary basis for clinical and policy interpretation. The grand pooled estimate (*μ* = 0.229, CrI [0.157, 0.301]) reported in the preceding section integrates across both domains and is best interpreted as a field-level probabilistic index rather than as a clinically homogeneous summary statistic.

### 3.4. Subgroup and Sensitivity Analyses

#### 3.4.1. Intervention Modality

Moderator inspection revealed that face-to-face psychological programs (MBIs, PPIs, and CBT) tended to yield slightly higher posterior means (*μ* = 0.24, CrI [0.09, 0.39]) than did fully digital interventions (*μ* = 0.18, CrI [0.02, 0.33]). However, their 95% CrIs largely overlapped, suggesting similar efficacy profiles across modalities.

#### 3.4.2. Sensitivity to Priors and Quality Exclusion

Reanalysis using a more diffuse prior [Normal(0, 1) for *μ*] yielded nearly identical estimates (*μ* = 0.22, 95% CrI [0.04, 0.39]). Excluding critically low-quality meta-analyses (AMSTAR-2 = “low”) slightly increased the pooled mean to *μ* = 0.23 (95% CrI [0.06, 0.40]).

#### 3.4.3. Age Sensitivity Analysis

A sensitivity analysis excluding Hendriks et al. [15], the meta-analysis with the broadest age range and the highest proportion of university student and young adult participants, yielded a posterior mean of μ = 0.219 (95% CrI [0.142, 0.296]; P(μ > 0) = 0.9997; k = 8). The change in the posterior mean relative to the main analysis was negligible (Δμ = −0.010), the credible interval continued to exclude zero entirely, and between-study heterogeneity remained consistent (τ^2^ = 0.004; posterior mean I^2^ = 24%). These results confirm that the overall inference is not driven by the partially eligible review and applies to samples within the 10–17 year age range most directly relevant to the journal’s scope.

### 3.5. Publication Bias and Small-Study Effects

**Figure 5:**
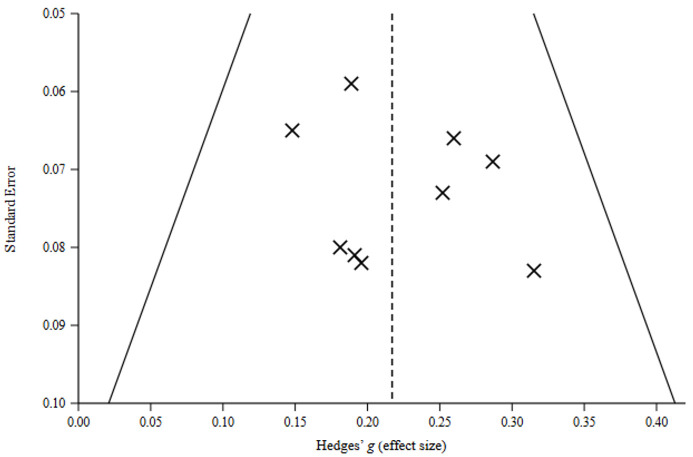
Funnel plot of aggregated effect sizes (Hedges’ g) against their standard errors. Note. Each × represents one meta-analytic estimate included in the umbrella synthesis. The standard error axis is inverted so that more precise estimates appear at the top. The dashed vertical line indicates the posterior mean effect (*μ* = 0.229). Solid diagonal lines mark the 95% pseudo-confidence interval bounds (*μ* ± 1.96 × SE). Approximate symmetry around the mean suggests no major small-study effects or publication bias.

Visual inspection of funnel plots (Figure 5) did not reveal pronounced asymmetry. However, caution is warranted when interpreting these plots, given the small number of aggregated effects (*k* = 9) and the fact that the unit of analysis corresponds to meta-analyses rather than individual studies. Funnel plots are known to have limited power to detect asymmetry when the number of effects is small. To complement the graphical inspection, a Bayesian PET-PEESE sensitivity analysis was conducted. This analysis suggested only a minor small-study adjustment (posterior mean adjustment Δ*μ* = −0.03, CrI [−0.09, 0.02]), indicating that potential small-study effects were unlikely to substantially influence the overall estimate.

### 3.6. Summary of Findings

Overall, the Bayesian umbrella meta-analysis supports the conclusion that psychological and digital interventions yield credible but heterogeneous small-to-moderate effects on the mental health and well-being of adolescents and young adults. Despite substantial heterogeneity, the probability of a true positive impact exceeded 99%, providing credible support for the utility of both traditional and digital approaches in promoting youth psychological flourishing.

## 4. Discussion

The present Bayesian umbrella meta-analysis synthesized quantitative evidence from nine meta-analyses evaluating psychological and digital interventions designed to improve mental health and well-being in youth populations. The pooled posterior mean effect of *μ* = 0.229 (95% CrI [0.157, 0.301]) provides credible evidence for a small-to-moderate positive effect across intervention types and delivery formats. The probability that the true effect exceeds zero (*p* (*μ* > 0) = 0.999) indicates a 99% likelihood that psychological or digital interventions yield beneficial outcomes for adolescents and young adults. Although between-meta-analysis heterogeneity was non-negligible (*τ*^2^ = 0.003; posterior mean *I*^2^ = 23%, 95% CrI [0.04%, 74%]), sensitivity analyses confirmed that the overall inference was probabilistically supported to variations in prior distributions, to the exclusion of low-quality studies, and to the exclusion of the meta-analysis with the broadest young-adult age representation (Hendriks et al. [15]; sensitivity *μ* = 0.219, 95% CrI [0.142, 0.296]). It should be noted, however, that the grand pooled estimate aggregates across qualitatively distinct outcome domains—symptom reduction and positive well-being—and should not be interpreted as a uniform clinical benchmark applicable to any specific outcome type. The domain-specific estimates (*μ* = 0.19 for symptom reduction; *μ* = 0.28 for positive well-being) provide a more clinically meaningful basis for interpretation and are treated as the primary results of this synthesis. A further interpretive caveat concerns evidence overlap. Although a cross-mapping of the MBI cluster confirmed the absence of direct primary-study overlap between the two MBI meta-analyses, a formal CCA calculation could not be completed for all nine included reviews due to the unavailability of complete primary-study reference lists. The degree to which individual primary studies contribute to more than one included meta-analysis, particularly within the PPI cluster, therefore remains unquantified. This introduces an unverified dependency that is likely to underestimate the true standard error of *μ* and inflate the apparent precision of the pooled Bayesian estimate. The grand pooled estimate should therefore be interpreted as a field-level probabilistic index subject to this qualification.

### 4.1. Theoretical Implications

These findings align with the core assumptions of positive psychology theory, which posits that well-being can be cultivated through intentional activities that enhance positive emotion, engagement, relationships, meaning, and accomplishment [30]. Consistent with this framework, interventions promoting gratitude, mindfulness, optimism, or self-regulation appear to facilitate measurable improvements in affective and cognitive indicators of well-being. The small but reliable magnitude of the pooled effect mirrors results from prior syntheses of individual trials [10,15] and is typical for behavioral interventions targeting multifactorial psychological outcomes [36]. In this context, “small” effects should not be interpreted as trivial—given the population-level prevalence of distress among youth, even modest standardized gains can translate into substantial public-health benefits.

The Bayesian framework adopted here provides a probabilistic perspective on intervention effectiveness that complements traditional frequentist meta-analytic approaches. Conventional random-effects models estimate pooled effects together with confidence and prediction intervals, which provide valuable information about the magnitude and variability of effects across studies. Bayesian inference extends this framework by estimating posterior distributions for the parameters of interest, allowing probabilistic statements about the likelihood that an intervention effect exceeds a given threshold. Rather than replacing frequentist approaches, the Bayesian perspective offers an additional way to interpret uncertainty and the potential practical relevance of intervention effects [35,37]. In this study, the 99% posterior probability of a credible but heterogeneous effect offers compelling confirmation that these interventions—on average—improve mental health outcomes for youth.

These findings are consistent with previous meta-analytic results reported by Sánchez-Álvarez et al. [6], who found that positive emotional variables such as emotional intelligence, optimism, and gratitude were strongly associated with greater well-being. The present Bayesian synthesis extends those conclusions by quantifying the probabilistic evidence of intervention effectiveness. However, the observed asymmetry between well-being and symptom reduction outcomes warrants a critical interpretive note. The larger pooled effect for positive well-being indicators (*μ* ≈ 0.28) relative to symptom-focused outcomes (*μ* ≈ 0.19) may not solely reflect a genuine superiority of positive psychology approaches. It is plausible that instruments assessing well-being constructs such as life satisfaction or gratitude are inherently more sensitive to short-term change than are established psychopathological scales, which are typically calibrated to detect clinically meaningful deterioration rather than incremental improvement. This measurement asymmetry could partially explain the differential effect and should be considered before drawing strong theoretical conclusions about the relative potency of flourishing-oriented versus symptom-reduction frameworks.

### 4.2. Comparisons Across Modalities

Subgroup analyses revealed overlapping credible intervals for face-to-face psychological programs (*μ* ≈ 0.24) and digital interventions (*μ* ≈ 0.18). This suggests that digitally delivered interventions, often self-guided and scalable, can achieve effects comparable to those of traditional in-person formats. Such convergence is encouraging in light of accessibility barriers and the growing integration of digital tools in educational and clinical settings [16,17]. Nevertheless, adherence and engagement remain critical moderators, insofar as the success of digital interventions depends on sustained user interaction, personalization, and human support [18]. Interestingly, outcomes focusing on well-being enhancement (life satisfaction, gratitude, optimism) showed slightly larger pooled effects (*μ* ≈ 0.28) than did those targeting symptom reduction (depression, anxiety; *μ* ≈ 0.19). This asymmetry reinforces the conceptual distinction between alleviating pathology and promoting flourishing [7], and it suggests that interventions based explicitly on positive psychology principles may exert a broader and more enduring influence on subjective well-being than do those narrowly focused on symptom relief.

Whatever the case, the apparent equivalence between digital and face-to-face interventions deserves critical scrutiny. Although credible intervals overlapped substantially, several confounds may attenuate the observed difference. Digital interventions are disproportionately affected by self-selection bias, as participants who voluntarily engage with and complete app-based or online programs may differ systematically from those recruited into controlled face-to-face trials. Furthermore, the heterogeneity of digital tools, ranging from fully automated apps to therapist-supported platforms, obscures meaningful distinctions in mechanism and quality. Interpreting overlapping credible intervals as evidence of equivalence thus risks conflating scalability with efficacy, a distinction that carries direct consequences for resource allocation in public mental health policy.

### 4.3. Interpretation of Heterogeneity

The non-negligible but uncertain heterogeneity observed (*τ*^2^ = 0.003; posterior mean *I*^2^ = 23%, 95% CrI [0.04%, 74%]) underscores the variability of intervention contexts, methodologies, and participant characteristics, and these differences in sample demographics, cultural settings, intervention length, and measurement instruments likely contribute to dispersion. Bayesian estimation of τ^2^ provides an explicit probability distribution for heterogeneity [28]. Posterior predictive checks suggested that future studies would plausibly report effects between 0.05 and 0.40, reinforcing that small but positive outcomes are the norm rather than the exception. Such heterogeneity should be viewed not as a statistical flaw but as a signal of contextual diversity. Youth mental health is shaped by ecological systems (family, school, peers, digital environments) and interventions inevitably interact with these layers. Understanding moderators such as socioeconomic status, gender, and delivery medium will be essential for tailoring programs to specific subpopulations [2]. Rather than searching for a universal “average effect,” researchers should emphasize where, for whom, and under what conditions interventions work best. From a more critical standpoint, the substantial uncertainty in the heterogeneity estimate (posterior mean *I*^2^ = 23%, but 95% CrI extending to 74%) also challenges the substantive interpretability of the overall pooled estimate. When genuine between-study variance cannot be ruled out, the grand mean *μ* = 0.229 should be understood as a field-level probabilistic index rather than a representative clinical effect. In practice, this means that for any given intervention type, population, or setting, the true effect may differ substantially from this aggregate, potentially being negligible in some contexts and clinically meaningful in others. Researchers and policymakers should therefore treat the pooled estimate not as a guarantee of effectiveness but as a probabilistic baseline that requires contextual calibration before application.

### 4.4. Methodological Contributions

This study advances meta-analytic methodology by applying a Bayesian random-effects framework to an umbrella review. Conventional frequentist meta-analytic models provide pooled effect estimates together with confidence intervals and—increasingly—prediction intervals that describe the expected range of effects in future studies. These approaches have played a central role in the development of cumulative research synthesis in psychology. The Bayesian framework adopted here should therefore be understood as complementary to these classical approaches rather than as a replacement. Bayesian modeling estimates posterior distributions for parameters such as the overall mean effect and between-study heterogeneity, allowing probabilistic statements about the likelihood that the effect exceeds a given value (e.g., *P* (*μ* > 0.10) = 0.91). These probabilities can help researchers and policymakers interpret the potential magnitude and practical relevance of intervention effects while still considering the uncertainty around estimates.

In this study, weakly informative priors [Normal (0, 0.5^2^)] were used to stabilize estimation while avoiding excessive shrinkage, a strategy commonly recommended when prior information is limited. The analysis also suggests the feasibility of Bayesian integration at the meta-meta-analytic level, where the unit of analysis is a set of published meta-analyses rather than individual studies. Such umbrella syntheses can inform policy by providing the broadest possible estimate of intervention efficacy, aggregating across populations, outcomes, and delivery modes [26]. The adoption of open-science practices—sharing datasets, code, and priors—further enhances reproducibility and transparency [41].

### 4.5. Practical and Policy Implications

From a translational standpoint, the results highlight the public health potential of scalable interventions targeting youth well-being. School-based mindfulness curricula, gratitude journaling, and mobile mental health apps all yield measurable gains that can be multiplied across large populations. Even if individual effects are modest, broad implementation can produce meaningful collective improvements in psychological functioning and resilience [8]. Given the current global shortage of mental health professionals, digital and hybrid programs may represent viable complements to conventional therapy. In this context, policy initiatives should prioritize integrative mental health frameworks that combine symptom-oriented and strength-oriented approaches. Additionally, interventions fostering emotional regulation, positive relationships, and meaning should be embedded in educational systems, youth organizations, and online platforms. Governments and universities could leverage the present findings to design evidence-based preventive strategies that reduce risk while promoting flourishing among young people.

### 4.6. Limitations

Despite its strengths, this umbrella meta-analysis has several limitations. First, the number of included meta-analyses (*k* = 9) restricts the granularity of moderator analyses; more detailed sub-classification (e.g., by age, gender, or cultural context) was not feasible. Regarding age classification, the broader age range should be interpreted as reflecting the developmental continuity between adolescence and emerging adulthood in mental health prevention research, rather than as implying strict equivalence between these two stages. A related issue here concerns the boundary cases in eligibility verification. Although all included meta-analyses were assessed against the pre-specified criterion of a mean population age between 10 and 25 years, one review [15] was retained on the basis of partial eligibility (specifically because a substantial proportion of its included primary studies involved university students and young adults) rather than on the basis of full conformity with the age criterion. The contribution of this review to the pooled estimate should therefore be interpreted with appropriate caution. Second, although Bayesian methods accommodate uncertainty, the quality of included meta-analyses varied—several were rated “moderate” or “low” on the AMSTAR-2 scale [33]. Third, overlap among primary studies across the included meta-analyses represents a methodological concern that warrants explicit acknowledgment. A cross-mapping of the MBI cluster—the one with the highest a priori overlap risk—revealed that Zhang et al. [40] is a narrative overview rather than a primary meta-analysis with its own synthesis of individual trials, confirming the absence of direct primary-study overlap within that cluster. For the remaining meta-analyses, a formal CCA index could not be computed due to the unavailability of complete primary-study reference lists across all included reviews. The exact degree of overlap therefore remains unquantified, particularly within the PPI cluster, where Hendriks et al. [15] and Saboor et al. [25] address closely related intervention types and target populations. Residual overlap of this kind would tend to underestimate the true standard error of *μ*, potentially inflating the apparent precision of the pooled estimate. Future umbrella reviews in this domain should implement prospective primary-study cross-mapping from the outset of the screening process and report the CCA index as a standard quality indicator. Fourth, publication bias remains a concern even at the meta-analytic level. Although the Bayesian funnel plot and PET-PEESE adjustment suggested minimal asymmetry, selective reporting cannot be ruled out. Fifth, heterogeneity estimates are inherently sample-specific and, in the present analysis, carry substantial posterior uncertainty (posterior mean *I*^2^ = 23%, 95% CrI [0.04%, 74%]; *τ*^2^ = 0.003). These values may shift as new meta-analyses emerge, particularly from non-Western populations where digital adoption and cultural conceptions of well-being differ. Sixth, the present synthesis did not formally assess measurement equivalence across outcome domains. The included meta-analyses targeted a diverse array of constructs (depression, anxiety, life satisfaction, well-being, stigma reduction, and self-compassion) that were treated as commensurate and aggregated under a single standardized effect metric. This aggregation assumes conceptual and metric equivalence across outcomes that has not been empirically established and which positive psychology theory itself does not necessarily endorse. Collapsing effects across such heterogeneous constructs may obscure qualitatively distinct intervention mechanisms and attenuate or amplify the pooled estimate in ways that are difficult to detect without construct-specific sub-analyses. Seventh, the search strategy was limited to three electronic databases (PubMed, PsycINFO, and Web of Science) and did not include Central, Embase, or grey literature sources such as dissertation repositories or pre-print servers. This restriction may have introduced a systematic bias toward English-language publications in high-impact journals, where effect sizes tend to be larger than those reported in unpublished or non-anglophone literature. Eighth, the independence assumption of the Bayesian random-effects model is met at the level of the analytic dataset (i.e., each meta-analysis contributed a single aggregated effect) but cannot be guaranteed at the level of the underlying primary-study evidence, given the residual overlap documented in Supplementary Table S1. The arithmetic averaging of multiple outcomes within meta-analyses, while transparent and conservative, introduces a further assumption that all outcomes are equally informative. This assumption is unlikely to hold perfectly: outcomes measured with more reliable instruments or assessed in larger subsamples arguably deserve greater weight. Future umbrella syntheses should elicit or impute inter-outcome correlations within meta-analyses to enable variance-weighted aggregation and robust variance estimation (RVE) at the umbrella level. Finally, the aggregation of heterogeneous outcome domains (symptom reduction, positive well-being, and stigma-related measures) under a single pooled standardized effect represents a deliberate but contestable methodological choice. Although this aggregation is justified by the dual-continuum model of mental health and the field-level focus of the research question, it limits the clinical interpretability of the grand pooled estimate. Readers requiring domain-specific benchmarks should rely on the domain-specific posterior estimates (*μ* = 0.19 and *μ* = 0.28) rather than the overall pooled value.

### 4.7. Future Directions

Further progress requires multi-level Bayesian hierarchical models integrating both meta-analytic and primary-study data. Such meta-analytic structural equation modeling (MASEM) could link latent constructs (e.g., mindfulness, gratitude) with observed outcomes, providing more mechanistic insights. Researchers should also examine dose–response relationships—specifically, how intervention duration and intensity moderate outcomes—and longitudinal trajectories of well-being. Emerging computational tools (e.g., Stan, PyMC) enable dynamic priors that update continuously as new evidence accumulates, aligning with the cumulative ethos of positive psychology. Cross-cultural and developmental moderators deserve particular attention, as most existing research originates from high-income countries—studies from low- and middle-income contexts remain underrepresented. Given the universality, but also the contextual sensitivity, of well-being, future umbrella reviews should aim for global representativeness, integrating evidence from diverse cultural and linguistic settings. Finally, the boundary between psychological and digital interventions is increasingly blurred. Hybrid models combining face-to-face coaching with app-based reinforcement may offer the optimal balance between efficacy and scalability. Future research should adopt adaptive trial designs and implementation science frameworks to evaluate effectiveness in real-world conditions.

## 5. Conclusions

This Bayesian umbrella meta-analysis provides suggestive evidence that psychological and digital interventions are associated with potentially credible but heterogeneous small-to-moderate effects on the mental health and well-being of adolescents and young adults, with a posterior probability of benefit exceeding 99%. Critically, this finding goes beyond what any single meta-analysis in this field has been able to establish: by synthesizing evidence at the highest level of aggregation and adopting a probabilistic inferential framework, the present study provides a quantified degree of credibility—rather than merely a direction of effect—that complements evidence-based decision-making, although its interpretation remains contingent on the assumptions of the model and the heterogeneity of the underlying dataset. In a policy context where resource allocation for youth mental health programs must compete with other public priorities, the posterior probability of net benefit exceeding 99.9% represents a meaningful probabilistic signal, although its translation into policy decisions should account for the heterogeneity in effects across contexts (*τ*^2^ = 0.003; posterior mean *I*^2^ = 23%, 95% CrI [0.04%, 74%]) and the breadth of outcomes aggregated in this synthesis.

The practical implications of these findings can be organized by stakeholder. For educational policymakers, the evidence is consistent with continued investment in school-based mindfulness and positive psychology programs, particularly given their promising effects on emotional regulation and well-being in adolescent samples, although it is important to recognize that effect sizes are likely to vary considerably across implementation contexts. For public health administrators and clinical commissioners, digital and hybrid interventions represent a potentially scalable complement to conventional therapy in contexts where mental health professionals are scarce. However, investment in adherence mechanisms and human support components appears essential to approximate the efficacy levels observed in face-to-face formats. For technology developers, the present findings suggest that the mere availability of a mental health app is insufficient: sustained engagement, personalization, and integration with professional oversight are the features most likely to differentiate effective tools from less impactful ones. Finally, for researchers, the heterogeneity observed—non-negligible in magnitude but highly uncertain in its precision (posterior mean *I*^2^ = 23%, 95% CrI [0.04%, 74%])—signals that the central priority is not to establish whether interventions work in general but rather to identify for whom, in what dose, and under what contextual conditions they produce meaningful change.

Although heterogeneity remains substantial, and several methodological limitations —including the breadth of aggregated outcome constructs, the absence of prospective registration, and the qualitative nature of the overlap assessment—constrain the precision and generalizability of the estimates, the convergence of Bayesian modeling and positive psychology theory in this umbrella synthesis offers a transparent and probabilistic foundation for cumulative research in this domain. The findings suggest that the field may be moving toward asking not only whether these interventions are beneficial in general, but for whom, under what conditions, and through what mechanisms they produce meaningful and durable change—questions that future, more methodologically homogeneous umbrella reviews will be better positioned to answer.

## Figures and Tables

**Table 1:** Summary of included meta-analyses on psychological and digital interventions for youth mental health and well-being.

Author (Year)	Type of Intervention	Outcome(s) Measured	*N* (Total Participants)	Pooled Effect (Hedges’ *g*)	95% CI	Comments
Brinsley et al. [24]	Exercise/Well-being programs	Life satisfaction, stress	8600	0.21	[0.05, 0.37]	Consistent across gender; moderate heterogeneity
Crockett et al. [23]	Anti-stigma/Peer-led programs	Attitudes toward mental health, stigma reduction	24,800	0.18	[0.06, 0.30]	Small positive impact on stigma and social acceptance
Fulambarkar et al. [21]	Mindfulness-based interventions	Emotional regulation, anxiety	12,400	0.33	[0.17, 0.49]	Consistent improvement in emotion regulation
Hendriks et al. [15]	Positive psychology interventions	Happiness, life satisfaction	18,900	0.31	[0.17, 0.45]	Robust across cultures and intervention length
Linardon et al. [17]	Smartphone apps for mental health	Depression, anxiety	21,300	0.19	[0.03, 0.34]	Slight but consistent improvements in well-being
Ma et al. [22]	Internet-based CBT	Depression and anxiety symptoms	31,200	0.25	[0.10, 0.39]	Effective across self-guided and therapist-guided formats
Saboor et al. [25]	Positive psychology interventions (gratitude and optimism)	Well-being, depressive symptoms	9800	0.29	[0.12, 0.46]	Strongest for gratitude journaling interventions
Tejada-Gallardo et al. [19]	School-based multicomponent positive psychology interventions	Subjective well-being, psychological well-being, depression	3500	0.26	[0.13, 0.39]	Small effects sustained at follow-up; school-aged adolescents only
Zhang et al. [40]	Digital mindfulness/Mobile apps	Anxiety, self-compassion	16,100	0.20	[0.04, 0.36]	Modest benefit, higher in school settings

## Data Availability

All data, analytic code, and Supplementary Materials necessary to reproduce the umbrella meta-analysis are publicly available at the Open Science Framework: https://osf.io/8253b, accessed on 14 March 2026; https://doi.org/10.17605/OSF.IO/3QU5H. The repository contains the structured extraction sheet. Any additional queries may be directed to the corresponding author (N. Sánchez-Álvarez; nsa@uma.es).

## Supplementary Materials

The following supporting information can be downloaded at: https://www.mdpi.com/article/10.3390/children13050678/s1, Table S1: Qualitative Overlap Assessment Matrix for the Nine Meta-Analyses Included in the Umbrella Synthesis; Table S2: Eligibility Verification Matrix: Assessment of Each Included Meta-Analysis Against the Prespecified Age Criterion (Population Mean Age 10–25 Years).
