# Supplementary material for: Meta-Analysis of Psychological and Digital Interventions to Enhance Mental Health and Well-Being in Youth: A Bayesian Umbrella Review
Source: Children (Basel). 2026 May 14;13(5):678. doi: 10.3390/children13050678 (PMC13204972; doi:10.3390/children13050678)
Supplement: Supplementary file 1 [file children-13-00678-s001.zip › children-4230628-supplementary.pdf]

Supplementary Table S1

Qualitative Overlap Assessment Matrix for the Nine Meta-Analyses Included in the Umbrella Synthesis

Part A. Characteristics of Included Meta-Analyses

Characteristic	Crockett et al, [23]	Fulambarkar et al. [21]	Ma et al. [22]	Brinsley et al. [24]	Saboor et al. [25]	Zhang et al. [38]	Tejada-Gallardo et al. [19]	Hendriks et al. [15]	Linardon et al. [17]
**Intervention domain**	Anti-stigma / peer-led	MBI (school)	Internet-based CBT	Peer-led / well-being	PPIs (digital)	Digital MBI / mobile apps	PPIs (multi-component)	PPIs (multi-component)	Smartphone apps
**Target population**	Youth 10–25 y	Adolescents 10–18 y	Adolescents / young adults	Adolescents 10–18 y	Children / young adults	Youth / adults	Adolescents	Adults / youth	Adults / youth
**Primary outcome**	Stigma / attitudes	Emotional regulation, anxiety	Depression, anxiety	Life satisfaction, stress	Well-being, depression	Anxiety, self-compassion	Happiness, well-being	Happiness, life satisfaction	Depression, anxiety
**AMSTAR-2 quality**	High	Moderate	Moderate	Moderate	Low	Moderate	Moderate	High	High
**No. primary studies (k)**	~85	~60	~120	~40	~45	~55	~80	~100	~65

Part B. Pairwise Qualitative Overlap Matrix

Meta-analysis	Crockett et al, [23]	Fulambarkar et al. [21]	Ma et al. [22]	Brinsley et al. [24]	Saboor et al. [25]	Zhang et al. [38]	Tejada-Gallardo et al. [19]	Hendriks et al. [15]	Linardon et al. [17]
**Crockett et al, [23]**	—	N	N	N	N	N	N	N	N
**Fulambarkar et al. [21]**	N	—	N	N	N	P	P	P	N
**Ma et al. [22]**	N	N	—	N	N	N	N	N	P
**Brinsley et al. [24]**	N	N	N	—	N	N	N	N	N
**Saboor et al. [25]**	N	N	N	N	—	N	P	P	N
**Zhang et al. [38]**	N	P	N	N	N	—	P	P	N
**Tejada-Gallardo et al. [19]**	N	P	N	N	P	P	—	P	N
**Hendriks et al. [15]**	N	P	N	N	P	P	P	—	N
**Linardon et al. [17]**	N	N	P	N	N	N	N	N	—

Legend

N = No overlap identified (distinct intervention domain or population, or no shared primary studies detected). **P** = Possible overlap (shared intervention domain and/or target population; shared primary studies cannot be excluded without full primary-study cross-mapping). — = Same meta-analysis (diagonal).

MBI = mindfulness-based intervention; **PPI** = positive psychology intervention; **CBT** = cognitive-behavioral therapy; **AMSTAR-2** = A Measurement Tool to Assess Systematic Reviews (version 2); k = number of primary studies reported in each meta-analysis.

Note. Overlap was assessed qualitatively based on the intervention domains, target populations, and inclusion criteria reported in each meta-analysis. Possible overlap (P) was assigned when two meta-analyses shared both intervention domain and population characteristics, indicating that they may have drawn from a common pool of primary studies. No formal cross-mapping of primary-study identifiers was conducted; the extent of actual shared primary studies therefore remains unquantified. This constitutes a methodological limitation acknowledged in the Limitations section of the manuscript. The Corrected Covered Area (CCA) index (Pieper et al., 2014) could not be calculated due to the absence of complete primary-study identifier lists across all included reviews. Overlap is most probable within the mindfulness-based intervention cluster (Fulambarkar, Zhang) and the positive psychology intervention cluster (Saboor, Hendriks, Tejada-Gallardo). These clusters were treated as distinct entries in the Bayesian umbrella synthesis because they differed sufficiently in delivery format, outcome measures, or population age range to warrant independent inclusion.

Supplementary Table S2

Eligibility Verification Matrix: Assessment of Each Included Meta-Analysis Against the Prespecified Age Criterion (Population Mean Age 10–25 Years)

Meta-analysis	Age criterion in original review	Mean age of included samples	Setting	Intervention type	Primary outcome domain	Eligibility decision and justification
**Crockett et al. [23]**	10–24 y (stated)	~18.7 y	School / university	Anti-stigma / peer-led	Stigma reduction, help-seeking attitudes	**Fully eligible**. Age range explicitly restricted to youth (10–24 y); exclusively school and university samples.
**Fulambarkar et al. [21]**	12–18 y (stated)	~14.5 y	School	Mindfulness-based (MBI)	Stress, depression, anxiety	**Fully eligible**. Exclusively school-based adolescent sample; mean age well within criterion.
**Ma et al. [22]**	Adolescents / young adults	~19 y	Mixed (school, community, clinical)	Internet-based CBT	Depression, anxiety	**Fully eligible**. Review explicitly targets adolescents and young adults; mean age within criterion.
**Brinsley et al. [24]**	Adolescents (stated)	~16 y	School / community	Peer-led well-being	Life satisfaction, perceived stress	**Fully eligible**. Peer-led interventions explicitly targeting adolescent school populations.
**Saboor et al. [25]**	Children, adolescents, young adults (mean age < 35 y)	~22 y	Digital / mixed	Digital PPIs	Well-being, ill-being	**Eligible**. Majority of included trials involve participants ≤ 25 y; review focused on young populations.
**Zhang et al. [38]**	Not age-restricted; samples predominantly youth/young adults	~18 y (median across included trials)	Mixed (school, community)	Digital mindfulness / mobile apps	Anxiety, self-compassion	**Eligible**. Pooled sample predominantly youth; review includes substantial school-based adolescent trials.
**Tejada-Gallardo et al. [19]**	10–18 y (stated)	~13 y	School	School-based multicomponent PPIs	Subjective well-being, psychological well-being, depression	**Fully eligible**. Exclusively school-based adolescent sample; age range explicitly restricted to 10–18 y.
**Linardon et al. [17]**	Not age-restricted; predominantly young adults	~25 y	Digital (smartphone apps)	Smartphone apps for mental health	Depression, anxiety	**Eligible (boundary case)**. Mean age at upper boundary of criterion (≈ 25 y); intervention primarily targets young adult populations.
**Hendriks et al. [15]**	Not age-restricted	Mixed (includes university students and adults)	Mixed (clinical, community, university)	Multicomponent PPIs	Subjective well-being, psychological well-being, depression	**Partially eligible (retained with caveat)**. Review not youth-specific; however, a substantial proportion of included primary trials involved university students and young adults (≤ 25 y). Retained on basis of partial eligibility; results should be interpreted with caution.

Note. Age criterion refers to the prespecified eligibility threshold for the present umbrella review: meta-analyses were eligible if the population they synthesized had a mean age between 10 and 25 years. Mean age of included samples is reported as the approximate mean or median across primary studies included in each meta-analysis, where available. **Fully eligible** = age range of included population is clearly within criterion. **Eligible (boundary case)** = mean age at or near the upper boundary of the criterion. **Partially eligible (retained with caveat)** = review not youth-specific but includes a substantial proportion of youth-aged primary studies; retained with explicit limitation noted. PPI = positive psychology intervention; MBI = mindfulness-based intervention; CBT = cognitive-behavioral therapy; y = years.
